# Involvement of Microbiota in Insect Physiology: Focus on B Vitamins

**DOI:** 10.1128/mbio.02225-22

**Published:** 2022-12-13

**Authors:** Javier Serrato-Salas, Mathilde Gendrin

**Affiliations:** a Microbiota of Insect Vectors Group, Institut Pasteur de la Guyane, Cayenne, French Guiana; b Department of Insect Vectors, Institut Pasteur, Université Paris-Cité, Paris, France; Yale University; Ohio State University

**Keywords:** B vitamins, developmental biology, insect, lifespan, metabolism, microbiota, reproduction, symbiosis

## Abstract

Insects are highly successful in colonizing a wide spectrum of ecological niches and in feeding on a wide diversity of diets. This is notably linked to their capacity to get from their microbiota any essential component lacking in the diet such as vitamins and amino acids. Over a century of research based on dietary analysis, antimicrobial treatment, gnotobiotic rearing, and culture-independent microbe detection progressively generated a wealth of information about the role of the microbiota in specific aspects of insect fitness. Thanks to the recent increase in sequencing capacities, whole-genome sequencing of a number of symbionts has facilitated tracing of biosynthesis pathways, validation of experimental data and evolutionary analyses. This field of research has generated a considerable set of data in a diversity of hosts harboring specific symbionts or nonspecific microbiota members. Here, we review the current knowledge on the involvement of the microbiota in insect and tick nutrition, with a particular focus on B vitamin provision. We specifically question if there is any specificity of B vitamin provision by symbionts compared to the redundant yet essential contribution of nonspecific microbes. We successively highlight the known aspects of microbial vitamin provision during three main life stages of invertebrates: postembryonic development, adulthood, and reproduction.

## INTRODUCTION

Insects are the broadest and most diverse clade in the animal kingdom. Like other animals, they are not able to produce some essential metabolites, including some amino acids, which are crucial building blocks of the organism and thus required in large quantities, and vitamins, which are nutrients required in small quantities as they catalyze central metabolic pathways. The insect’s ability to occupy a great diversity of ecological niches largely depends on the variability of their diet and on the beneficial microorganisms that they harbor, which provide them with essential nutrients lacking in their diet (1–3). Host-microbe relationships in insects influence diverse aspects of their physiology related to digestion, nutrition, defense against pathogens, behavior, immunity, detoxification, reproduction, and renewal of the intestinal epithelium (4–7). The study of the nutritional contribution of microbes to insect development started in the first half of the 20th century with the identification of minimal nutritional requirements of insects in sterile conditions. Early research suggested an essential role of bacteria in provisioning amino acids and B vitamins, thereby supporting the development and survival of various insects (8–10). More recent data were overall in line with such observations, yet also reported inconsistencies where previously reported diets may not support full development in the complete absence of bacteria (11). Methodological errors such as dietary impurities or undetected microbial contamination in germfree individuals may have undermined the importance of specific microbe-derived nutrients (10). Alternatively, some vitamins may have not been deemed essential because they are required in minute amounts, hence present in sufficient quantity in the egg to complete development (12). The improvement of techniques in molecular biology, chemistry, and high-throughput sequencing facilitates more precisely controlled experimental conditions and the possibility of combining experimental work with genomic analyses in order to produce clearer data on nutritional contributions of microbiota members to their insect hosts (1).

Our current knowledge of host-microbe nutritional interactions is mainly focused on host-bacterium relationships, even though there is also some evidence of nutritional symbiosis with yeasts and viruses (13–15) and of nutritional competition between hosts and eukaryotic parasites (16–18). Among the insect-associated bacteria, endosymbionts are intracellular bacteria that are vertically transmitted from mother to offspring in the egg cytoplasm. Among them, *Wolbachia* is an alphaproteobacterium colonizing 40% of insect species; *Wigglesworthia* and *Buchnera* are two *Gammaproteobacteria* found in tsetse flies and aphids, respectively, carried in a specific organ called the bacteriocyte; several *Spiroplasma* bacterial species, which belong to *Mollicutes*, colonize 5 to 10% of insect species and have extracellular and intracellular forms (19–22). Endosymbionts generally have a reduced genome size characterized by increased A-T content and gene loss (23, 24). These features may be explained by a combination of two evolutionary biology models. First, Muller’s ratchet hypothesis states that the constant evolution of organisms produces mutations, which accumulate and become costly to repair, triggering the loss of nonessential features. Second, the Black Queen hypothesis states that in the evolution of a community where resources may be shared, there is reduced selection pressure on maintenance of essential metabolic pathways in each member, resulting in reduced genome size and enhanced interdependence of community members (25, 26). In line with these genomic features, nutritional interactions have been reported between hosts and endosymbionts and have been classified as primary and secondary symbioses, whether the bacterium is essential or not to host physiology, respectively (27, 28).

Insects also harbor ectosymbionts, extracellular bacteria with a facultative association with their host, yet with closely related genome characteristics to endosymbionts, including genome reduction, gene loss, and signatures of horizontal gene transfers from/to genomes of the host or other microbes (2, 5, 23). The microbiota of insects also encompasses bacteria that do not have any specific association with their host; they may contribute to host nutrition but can be replaced by almost any other bacterium able to colonize the host (29). Some of these bacteria cannot strictly be defined as extracellular or intracellular. In addition to the above-mentioned example of *Spiroplasma* endosymbionts, the known extracellular bacteria Cedecea neteri and Serratia marcescens have been reported to invade cells of mosquito tissues and to replicate intracellularly (30). Here, we review the current knowledge on B vitamin provision by the microbiota to its insect host, considering as “microbiota” any microbe hosted by the insect, whether strictly symbiont or not. In line with the literature, a large part of this review focuses on bacteria. Since ticks share a similar life cycle with some insects and harbor similar bacteria as some insects, we also included tick-microbe interactions in our review. Indeed, we hypothesized that a wide focus on different types of diets and of host-microbe associations would allow us to see the big picture on conserved and specific features of host-microbe nutritional mutualism. By gathering information and cross-analyzing the role of B vitamins in various relationships, we successively highlight their importance in (i) fostering postembryonic development, (ii) affecting adult life span and homeostasis, and (iii) supporting reproduction (see Box 1).

BOX 1: VITAMINS, VITAMERS, AND KNOWN METABOLIC FUNCTIONS

B vitamins are a group of water-soluble micronutrients that can act as cofactors of diverse metabolic processes in the cell. Each vitamin can be present in different forms, referred to as vitamers, the chemical structures that can complement each other to perform its biological activity (1). Insects generally cannot produce B vitamins themselves (31). This box describes the different B vitamins and indicates which taxa can produce them.

Thiamine (B_1_, C_12_H_17_N_4_OS) is a cofactor required for the biosynthesis of acetyl coenzyme A from pyruvate, thus linking glycolysis to tricarboxylic acid cycle (TCA). Therefore, it has essential roles in cellular bioenergetic processes leading to ATP production, as well as in the metabolism of glucose, amino acids, and lipids. It is biosynthesized by bacteria, plants, fungi, and archaea.

Riboflavin (B_2_, C_17_H_20_N_4_O_6_) is a precursor of flavin adenine dinucleotide (FAD) and flavin mononucleotide (FMN), essential cofactors of enzymes that belong to the electron transport chain, the TCA cycle and β-oxidation of fatty acids. They are biosynthesized by bacteria, plants, fungi, and archaea.

Niacin, nicotinamide, and nicotinic acid are the B_3_ vitamers (C_6_H_5_NO_2_), all precursors of NAD (NAD), a coenzyme involved in cellular redox balance reactions, the TCA cycle, the electron transport chain, and the synthesis of lipids and nucleic acids. It is biosynthesized by animals, plants, fungi, and bacteria.

Pantothenate, or pantothenic acid (B_5_, C_9_H_17_NO_5_), is a precursor of coenzyme A (CoA). As such, it is an essential factor for the TCA cycle and fatty acid oxidation. Panthenol and pantetheine are two B5 vitamers. All three B5 vitamers are biosynthesized by bacteria, plants and fungi.

B_6_ (C_8_H_11_NO_3_) exists in six main forms, namely, pyridoxine, pyridoxal and pyridoxamine (and their respective phosphorylated forms), pyridoxine 5′-phosphate, pyridoxal 5′-phosphate, and pyridoxamine 5′-phosphate. Pyridoxal 5′-phosphate is the main active form, which is an important cofactor for more than 140 enzymes, transaminases, methionine catabolism as cystathione synthase and cystathionase, glycogen phosphorylase and biosynthesis of sphingolipids, among the most relevant. They are biosynthesized by archaea, bacteria, protozoan, fungi, and plants.

Biotin [d-(+)-biotin, B_7_, C_10_H_16_N_2_O_3_S] is a cofactor for several carboxylases, especially acetyl coenzyme A (acetyl-CoA) carboxylase involved in fatty acid synthesis, pyruvate CoA carboxylase in gluconeogenesis, β-methyl-crotonyl CoA carboxylase in leucine degradation, and propionyl CoA carboxylase in amino acid and fatty acid degradation. It is biosynthesized by plants, fungi, and bacteria.

Folate (B_9_, C_19_H_19_N_7_O_6_) refers to folic acid and its related compounds, notably its main active form tetrahydrofolate (THF). It is a methyl (one carbon) donor which plays a central role in the metabolism of nucleic acids and amino acids. It is biosynthesized by bacteria, fungi and plants.

Cobalamin (B_12_, C_63_H_88_CoN_14_O_14_P) is present in several forms, methyl-, hydroxy-, and adenosyl- and cyano-cobalamin, which act as coenzymes of isomerases, methyltransferases, or dehalogenases. It is notably involved in the breakdown of amino acids “fueling” the citric acid cycle and in the synthesis of methionine and of THF (thus involved in the metabolism of proteins and nucleic acids). It is biosynthesized by a few bacteria and archaea (1, 24, 32–34).

## INVOLVEMENT OF B VITAMINS DURING DEVELOPMENT

The life cycle of an insect usually begins with an egg covered by a resistant shell which contains its own nutrient reserves to develop until structures are in place for the hatching process to begin. Thereafter, immature mobile individuals will consume large amounts of food relative to their size to sustain their nutritional status as they move from one stage to the next, growing and gaining weight. The majority of immature arthropods are completely independent in foraging their own nutrients, except in a few viviparous species such as tsetse flies. Endosymbionts are inherently acquired through direct vertical transmission into the egg. The other microbiota members are acquired from the environment and via vertical transmission, as they contaminate the egg external envelope and are ingested when the immature individuals start to feed (35).

## BLOOD-FEEDING IMMATURES

**FIG 1:**
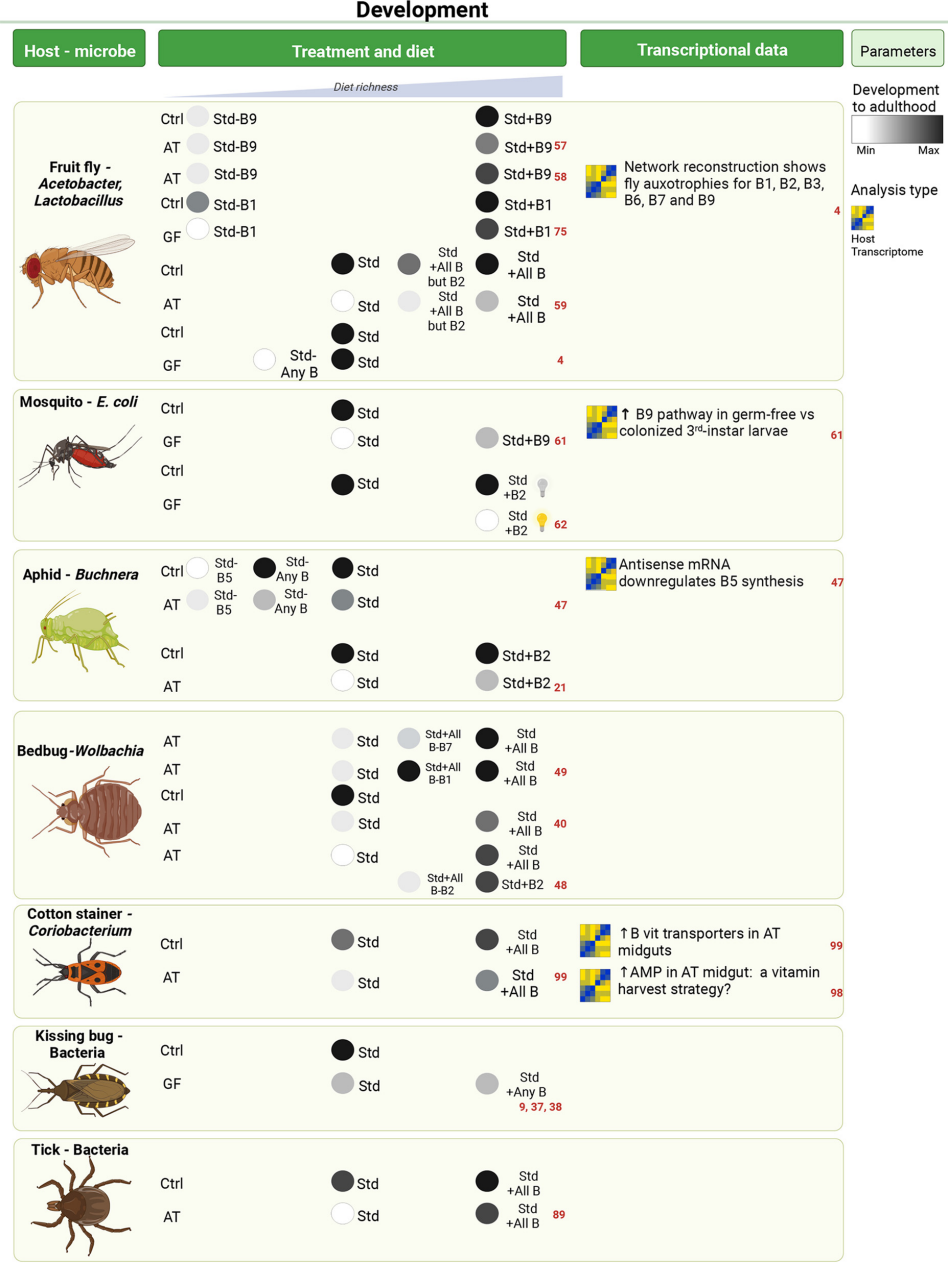
Influence of B vitamins on insect development (4, 9, 21, 37, 38, 40, 47–49, 57–59, 61, 62, 75, 89, 98, 99). The host column indicates the host reported in the corresponding study. These couples of hosts and microbes generally represent natural interactions except in the case of mosquitoes, where E. coli is used as a model bacterium. AT, antibiotic treated; GF, germfree; Ctrl, microbiota control (which can either mean conventionally reared or gnotobiotic, with AT and GF, respectively); Std, standard diet, neither impoverished nor enriched (a holidic diet is considered standard when it contains all the requirements for normal physiology). The image was created with BioRender.

**FIG 2:**
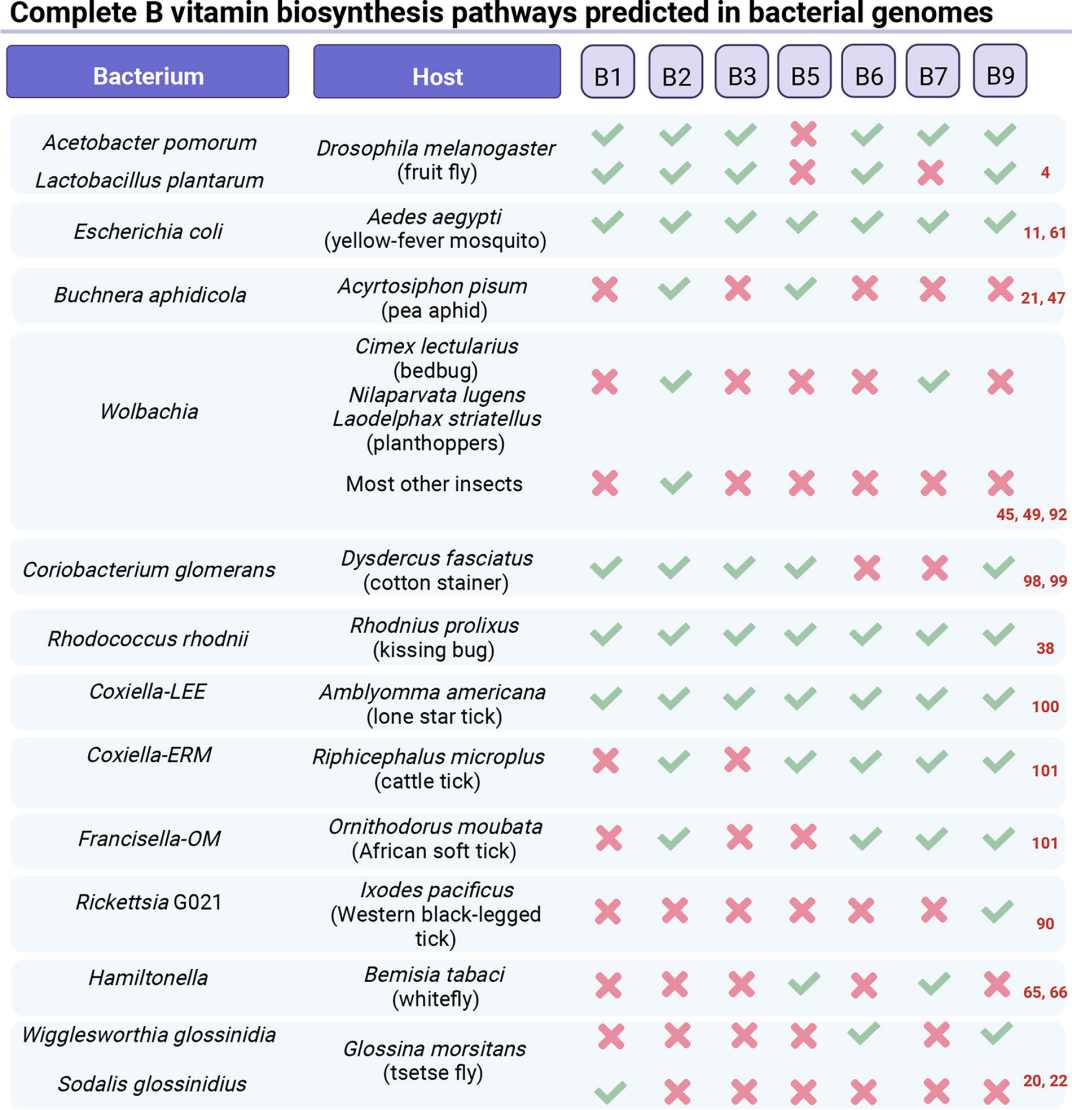
Predicted complete B vitamin biosynthesis pathways reported in some bacteria colonizing insects (4, 11, 20–22, 38, 45, 47, 49, 61, 65, 66, 90, 92, 98–101). The host column indicates the host reported in the corresponding study, whether it is a specific host-microbe association or not. A red “x” represents either the absence of the pathway or an incomplete pathway, while a green checkmark represents the presence of a complete pathway in the corresponding bacterial genome. Bacterium-host couples are ordered as they appear in Fig. 1, 3, and 4. The image was created with BioRender.

B vitamins have been found to be important in the microbiota-host interactions of obligate blood feeders (Fig. 1), as the blood is poor in B vitamins compared to what is generally required for insect development (1). Kissing bugs, *Rhodnius prolixus*, notably require *Rhodococcus* bacteria for nymph development, but the addition of B vitamins in the diet can rescue nymph development in the absence of *Rhodococcus* (9, 36, 37). More generally, an antibiotic treatment of immature bedbugs, kissing bugs or ticks leads to development delay, arrest, and/or death, and such effects can at least partly be rescued by the addition of a mixture of B vitamins (38–40). These experimental observations were corroborated with genomic analyses, suggesting that such symbionts can produce B vitamins (Fig. 2). Notably, tick-associated endosymbionts of the *Francisella*, *Coxiella*, *Arsenophonus*, and *Rickettsia* genera have reduced genomes compared to free-living species but keep intact B vitamin biosynthesis pathways (39, 41). A predicted ability for B vitamin production has also been reported in the genomes of kissing-bug-associated extracellular symbionts *Rhodococcus* and *Dickeya* (38, 42). In *Cimex* and *Paracimex* bedbugs, *Wolbachia*-cured nymphs can only develop if they receive riboflavin and biotin supplements. This is quite an exception in arthropods, where the colonization success of *Wolbachia* is generally linked to reproductive manipulation rather than to nutritional symbiosis. In these species, *Wolbachia* behaves as a primary endosymbiont; it is located in a bacteriocyte and biosynthesizes riboflavin and biotin (40, 43–45).

## SAP-FEEDING IMMATURES

Sap-feeding insects have also been found to rely on microbial B vitamins for their postembryonic development. This has specifically been demonstrated in several species of hemipterans, including aphids, planthoppers and leafhoppers (Fig. 1). Many aphid species harbor *Buchnera*, a primary endosymbiont present in a bacteriocyte and able to produce riboflavin (21). Removing this symbiont via antibiotic treatment delays the development of aphids fed on plants (46) and impedes development of nymphs fed on a riboflavin-deprived diet (47–49). However, a recent study showed that when curing 2-day-old larvae from *Buchnera*, aposymbiotic aphids (i.e., symbiont cured) had reduced developmental success compared to their symbiotic controls, yet their developmental success was not affected by any deprivation in B vitamins (47). Intriguingly, dietary pantothenate appeared critical for aposymbiotic and symbiotic aphid development, while the genome of *Buchnera* encodes a full pantothenate biosynthesis pathway (Fig. 2). A transcriptome analysis showed that *Buchnera* strongly expresses an antisense RNA in the *panC* and *coaE* genes, which encodes enzymes involved in pantothenate biosynthesis and pantothenate conversion to CoA, respectively. Accordingly, their proteins are undetectable, suggesting that these antisense RNAs repress the production of pantothenate and CoA in *Buchnera*. Functional implications of this repression are unclear.

Some aphid species additionally host other secondary endosymbionts such as *Spiroplasma*, *Rickettsia*, *Erwinia*, and *Wolbachia*, but their colonization success is related to reproductive manipulation rather than to nutritional mutualism (50). In contrast, some planthoppers harbor mutualist *Wolbachia*; as mentioned above for bedbugs, *Wolbachia* is present in a bacteriocyte, and cured immatures have a limited ability to reach adulthood unless receiving riboflavin and biotin supplements (40, 48, 49, 51). Similarly, the *Arsenophonus* endosymbiont of the date palm leafhopper *Ommatissus lybicus* is required for nymph development and its genomic sequence suggests that it can biosynthesize B vitamins (Fig. 2) (52, 53). *O. lybicus* also harbors a *Wolbachia* strain that is very similar to planthopper *Wolbachia* and is predicted to biosynthesize biotin. Moreover, individuals that lack *Arsenophonus* always carry *Wolbachia* (52–55). Hence, *Wolbachia* and *Arsenophonus* may be two alternative nutritional mutualists promoting development in *O. lybicus*. While the success of *Wolbachia* in arthropods is generally linked to reproductive manipulation rather than to nutritional symbiosis, we have cited several examples of hemipterans carrying a mutualist *Wolbachia* endosymbiont (bedbugs, plant hoppers, and potentially leafhoppers). Whether a mutualistic role is more widely spread in this order of insects is unclear, but *Wolbachia* symbionts appear to be slightly more prevalent in hemipteran species (69%) than overall in terrestrial insects (50%) (56).

## IMMATURES FEEDING ON MICROBE-CONTAINING DIETS

The importance of microbial B vitamins has been investigated in dipterans, including *Drosophila* and mosquitoes (Fig. 1), whose larvae develop on rotting fruit and in standing water, respectively. A standard laboratory *Drosophila* diet, based on yeast extract and corn meal, provides enough vitamins to allow full development of *Drosophila* larvae even in sterile conditions. Yet, this yeast-containing diet is already rich in microbe-derived vitamins. Hence, impoverished diets have been used to analyze the role of the microbiota in B vitamin provision. Notably, antibiotic-treated *Drosophila* larvae require dietary folate for successful development while conventionally reared larvae do not (57, 58). A further requirement on microbially sourced riboflavin and pantothenate was shown by diet manipulation in conventional and germfree flies (59). Finally, a recent study thoroughly assessed the role of the microbiota in the provision of 50 single nutrients, including B vitamins, by rearing gnotobiotic and germfree *Drosophila* larvae on 50 chemically defined diets, each deficient for a specific nutrient. This study showed that Acetobacter pomorum and Lactobacillus plantarum are both able to provide larvae with thiamine, riboflavin, nicotinic acid, biotin, and folate, which are all essential for larval development. A. pomorum is additionally able to support development in the absence of pyridoxine. None of these bacterial strains can support development in the absence of pantothenate, even though the growth of A. pomorum is able to grow without this vitamin (4). In a second study, these authors showed that both bacteria together are able to support larval development in the absence of pantothenate. A. pomorum provides pantothenate and biotin to *L. plantarum* which feeds A. pomorum with lactate. These bacteria enhance each other’s growth and provide nutrients for larval development (60). Considering mosquitoes, microbes are required for normal development (29), yet it is possible to produce adults with specific diets and rearing conditions, indicating that the microbiota contributes to larval nutrition (11). Our laboratory has recently set up a transient bacterial colonization system to investigate the role of bacteria during Aedes aegypti larval development (61). Larvae are colonized with bacteria which are auxotrophic for some bacterium-specific amino acids. As long as these amino acids are present in larval food, bacteria proliferate and support larval development. Bacteria are rapidly lost in the absence of these amino acids. When decolonizing larvae at the middle of larval development, the folate pathway is strongly upregulated and development rate is reduced, suggesting that bacterial folate participates in mosquito development. Accordingly, dietary supplementation in folate partly rescued the end of development of germfree larvae. Decolonization also leads to a defect in amino acid storage and in lipid incorporation in tissues, yet their link with folate or any other microbial metabolite is not established. Wang et al. further explored the nutritional requirements of germfree larvae by diet manipulation. A holidic (i.e., chemically defined) diet supplemented with commercial bovine lactalbumin supported development of germfree larvae in the dark, while removing riboflavin, pyridoxine, thiamine, or folate from this diet reduced development success. In contrast, removing pantothenate, nicotinic acid, or biotin had no impact (62). It is yet unclear whether these B vitamins are also essential for mosquitoes, as their presence or absence in commercial bovine lactalbumin has not yet been documented. Focusing on riboflavin, the same study showed that a *ribC*-deficient E. coli mutant, which cannot produce riboflavin, does not support larval development. The absence of riboflavin can be complemented by FAD and/or FMN, but not by its light-degradation product, lumichrome, consistent with the fact that the holidic diet with bovine lactalbumin cannot support development with normal light/dark cycle.

Hence, B vitamin metabolism is a focal point of bacterium-insect interactions during postembryonic development. Microbe-derived vitamins are provided either by vertically transmitted symbionts or by unspecific microbiota species and affect several parameters, including survival of immatures, development success and development speed. They are required for the development of immatures on vitamin-poor diets such as blood and sap. In the latter case, the microbiota plays the role of an insurance, enabling immatures to face nutritional constraints from the environment. The microbiota can notably be seen as a continuous source of vitamins, while dietary vitamins are degraded over time, in particular when exposed to light.

## INVOLVEMENT OF B VITAMINS IN ADULT PHYSIOLOGY AND SURVIVAL

The adult stage is particularly studied in insects because of its numerous implications in terms of ecology, agriculture and human health: insect species account for a number of pollinators, nutrient recyclers, soil caregivers, predators and preys, seed dispersers, crop pests, and pathogen vectors. In adults, the insect microbiota commonly behaves as commensal but can additionally participate to the digestion of recalcitrant diets, provision of micronutrients and production of short-chain fatty acids. A normal density of microbiota induces a basal activity of antimicrobial peptides and epithelial tissue turnover, whereas an imbalance due to the presence of pathogens (viruses or bacteria) and/or induced by ageing may lead to increased induction of basal immune responses with the production of reactive oxygen species and antimicrobial peptides and to increased cell proliferation (5, 63, 64).

**FIG 3:**
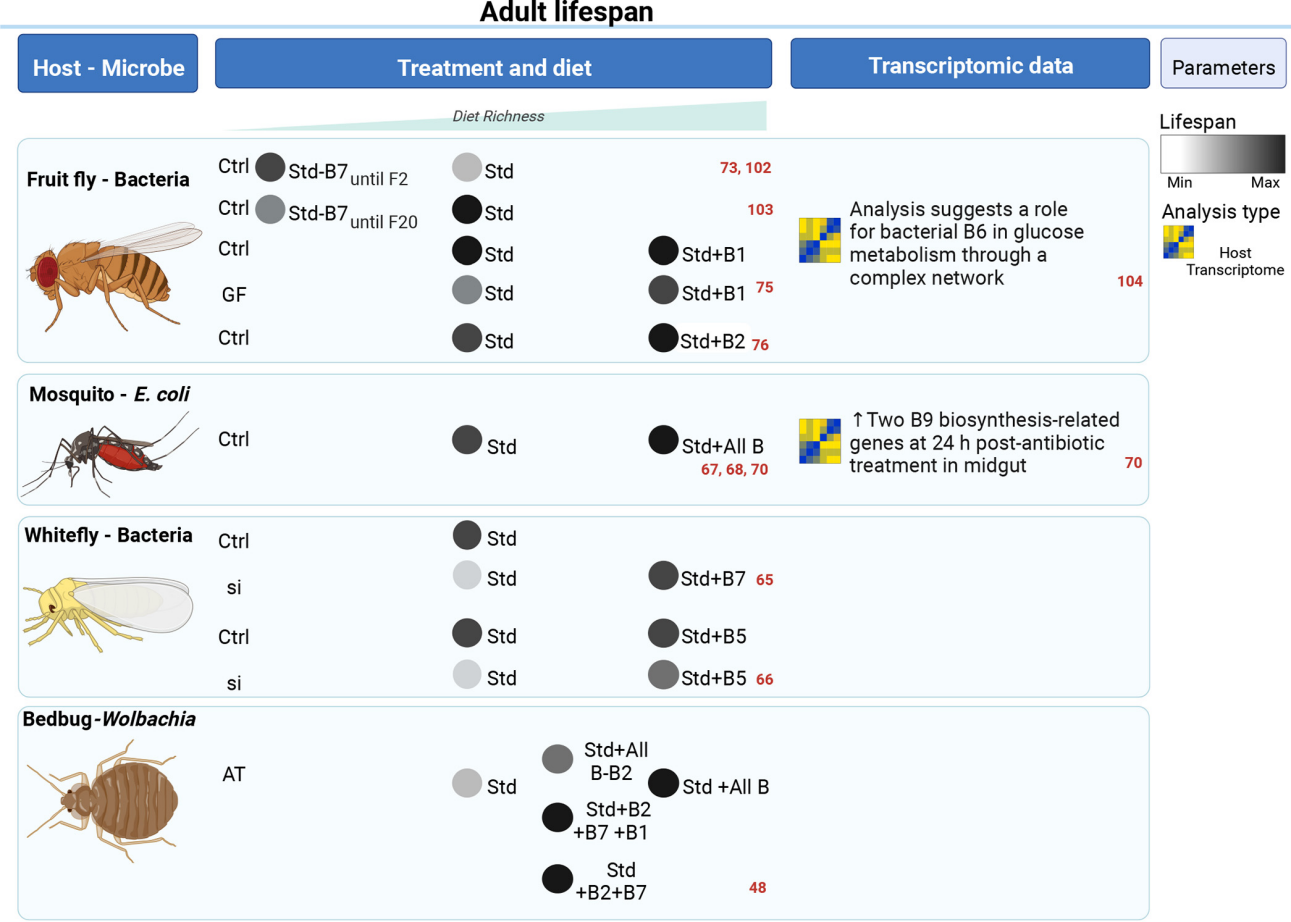
Influence of B vitamins on insect life span (48, 65–68, 70, 73, 75, 76, 102–104). AT, antibiotic treated; GF, germfree; Ctrl, microbiota control (which can either mean conventionally reared or gnotobiotic, with AT and GF, respectively); Si, gene silencing by double-strand RNA injection; Std, standard diet, neither impoverished nor enriched (a holidic diet is considered standard when it contains all the requirements for normal physiology). The image was created with BioRender.

When specifically focusing on the role of the microbiota as a provider of B vitamins, a standard readout for fitness is adult life span, but other quantified parameters also include gene expression, resistance to stress, symbiont density, or B vitamin levels. Four relevant examples of microbiota contribution in vitamin provision to the adult hosts are reported in this section and in Fig. 3. Reproductive phenotypes are reviewed in the next section as they affect the offspring and yet are largely influenced by the parental metabolism.

## IMPACT ON ADULT LIFESPAN

Among hemipterans, *Wolbachia* is essential to adult survival in the bedbug *Cimex lectularius*. The elimination of this symbiont leads to mortality of the bedbugs, which recovers its fitness when a B vitamin cocktail containing riboflavin and biotin is added to the blood diet (48). The case of phytophagous hemipterans, notably Bemisia tabaci whiteflies, reveals a particularly strong symbiotic integration between the host and its *Portiera* and *Hamiltonella* symbionts for pantothenate and biotin synthesis, respectively. First, the genome of *B. tabaci* includes *bioA*, *bioD*, and *bioB*, which encode functional enzymes of the biotin biosynthesis pathway (65). These horizontally transferred genes have similar sequences to their *Wolbachia*, *Cardinium*, and *Rickettsia* orthologs and are also present in *Hamiltonella* (Fig. 2). An antibiotic treatment specifically targeting *Hamiltonella* leads to a reduction of biotin titers while whitefly *bioA*, *bioD* and *bioB* are induced in bacteriocytes. Silencing any of these host genes increases female mortality, unless diet is supplemented with biotin. Second, the whitefly genome encodes *panBC*, a gene with a similar sequence to two genes of the pantothenate biosynthesis pathway in Pseudomonas, *panB* and *panC* (66). Among whitefly symbionts, *Portiera* lacks these genes in the pantothenate biosynthesis pathway and in *Hamiltonella* and *Rickettsia* this pathway is completely absent (Fig. 2). A rifampicin treatment of whiteflies leads to the elimination of the three main symbionts, reduces pantothenate levels, and tends to increase mortality in adult females. Silencing of *PanBC* expression also reduces *Portiera* abundance, pantothenate level and female life span, which can be partly rescued with pantothenate supplements. Together, these studies indicate that *B. tabaci* cooperates with *Hamiltonella* to produce biotin and with *Portiera* to produce pantothenate, using genes acquired from bacteria via horizontal gene transfers.

With regard to mosquitoes, adult colonies are maintained with sugar solutions, in which multivitamin syrups are sometimes added to improve fitness. Such diet supplementation notably enhances the life span of *Anopheles* and *Culex* mosquitoes, and this effect even remains in *Culex* until the next generation (67, 68). Conversely, an antibiotic treatment has been found to reduce the life span of *Anopheles* mosquitoes, which can be recovered after reintroduction of *Serratia* and Enterobacter. In *Aedes*, a positive impact of colonization with Escherichia coli on adult life span has also been detected, but only if larvae have been grown in axenic conditions: colonization of adults with E. coli did not impact life span compared to germfree adults produced by transient colonization (11, 61), suggesting that this positive impact of the microbiota depends on larva-to-adult carryover effects. Along these lines, adults originating from larvae carrying a conventional microbiota generally have a longer life span than adults coming from monocolonized larvae (69). Whether the positive impact of the microbiota on adult life span is due to vitamin provision needs to be investigated. A transcriptomic analysis detected an enrichment in the folate biosynthesis pathway in antibiotic-treated blood-fed *Anopheles* mosquitoes, suggesting that the microbiota may well have an important role in B vitamin provision to adults (70). Such an impact of the microbiota after the blood meal may be linked to bacterial biosynthesis and/or to a participation in harvesting vitamins present in red blood cells via hemolysis and in microbes via antimicrobial activity (71, 72).

In fruit flies, the specific activities of thiamine, riboflavin, and biotin have been analyzed. Biotin deficiency in the diet decreases life span in males and females but increases resistance to stress in males (73). This vitamin is particularly important for mitosis of intestinal stem cells. When these cells are unable to import biotin, they do not proliferate properly, which notably increases the susceptibility of flies to bacterial infection. Flies fed with a biotin-deficient diet have a lower mitosis rate in the intestine, but Escherichia coli colonization can restore it to normal levels (74). Thiamine deficiency in the diet does not seem to affect adult life span whether in conventionally reared or axenic individuals (75). Riboflavin has been proposed as an anti-aging agent, since conventionally reared flies supplied with additional riboflavin in the diet have a prolonged life expectancy in normal conditions and upon oxidative stress (76), but it is not clear whether riboflavin has to be provided by the microbiota in adults.

In addition to the examples described in Fig. 3, antibiotic treatment leads to a decrease in adult life span in several other insect species, where bacteria have been found to carry genes coding B vitamins biosynthesis pathways. This has notably been found in date palm leafhoppers *Ommatissus lybicus* carrying *Wolbachia* (52, 53, 55) and in hematophagous flies *Hippoboscoidea*, *Streblidae*, and *Nycteribiidae* carrying several *Arsenophonus*, *Sodalis*, and/or *Aschnera* (77–79).

## IMPACT ON OTHER LIFE HISTORY TRAITS IN ADULTS

Provision of microbial B vitamins to the host has also been found to affect traits other than life span. A recent ecological study indicated that an invasion by Solenopsis invicta ants induces a change of diet of the endemic ant workers *Tapinoma melanocephalum* toward a protein-rich diet, which affects microbiota composition, notably increasing relative abundance of *Wolbachia* (80). This study further shows that supplementing the diet of *T. melanocephalum* in noninvaded and invaded colonies with protein and sugar, respectively, reverses the effect on several taxa, including *Wolbachia*, and leads to a loss of riboflavin and nicotinic acid in invaded ants, whereas the concentrations of some other B vitamins are not affected. This suggests that the change in diet may allow ants to behaviorally respond to invasion by favoring a microbiota producing riboflavin and nicotinic acid.

Considering vectorial transmission, the impact of microbe-derived vitamins on vector competence has been less extensively studied, though there is evidence to suggest its importance in tsetse flies (18). Trypanosome infections induce folate biosynthesis genes of the fly symbiont *Wigglesworthia*. In the major trypanosome vector Glossina morsitans, an antifolate treatment decreases parasite infection prevalence in the gut, without affecting subsequent parasitic development toward salivary gland infection. In the inefficient vector *Glossina brevipalpis*, diet supplementation in folate leads to a strong increase in trypanosome infection prevalence. Finally, folate gene expression by *Wigglesworthia* is higher in Glossina morsitans than in less-efficient vectors and induced by parasitic infection, suggesting that folate expression may be a critical factor determining vector competence toward the parasite. In other vectors, a link with the microbiota is less clear, but there is evidence that parasites require B vitamins from the host and/or the environment. Diet supplementation with folic acid or one of its precursors, *para*-aminobenzoate, positively affects Brugia malayi development in *A. aegypti* (81). Genetic studies have found that pantothenate transporter and predicted pantothenate kinases Pank1 and Pank2 are required for *Plasmodium* development in the mosquito, while they are dispensable for growth in red blood cells and differentiation to gametocytes (16, 82).

## B VITAMINS AND REPRODUCTION

Arthropod reproduction is a highly demanding process in terms of energetic investment, macromolecule synthesis, and accumulation. It involves several processes, including oogenesis, spermatogenesis, copulation, and embryogenesis, ultimately leading to hatching of viable larvae. Typical readouts of reproduction are fertilization success, the proportions of egg-laying females and the sizes of their clutches, and the egg hatching rate. In addition, the viability of larvae and the sex ratio of the resulting adult offspring may be considered parameters of reproduction if the focus of the study is the treatment of the parents (83–85).

**FIG 4:**
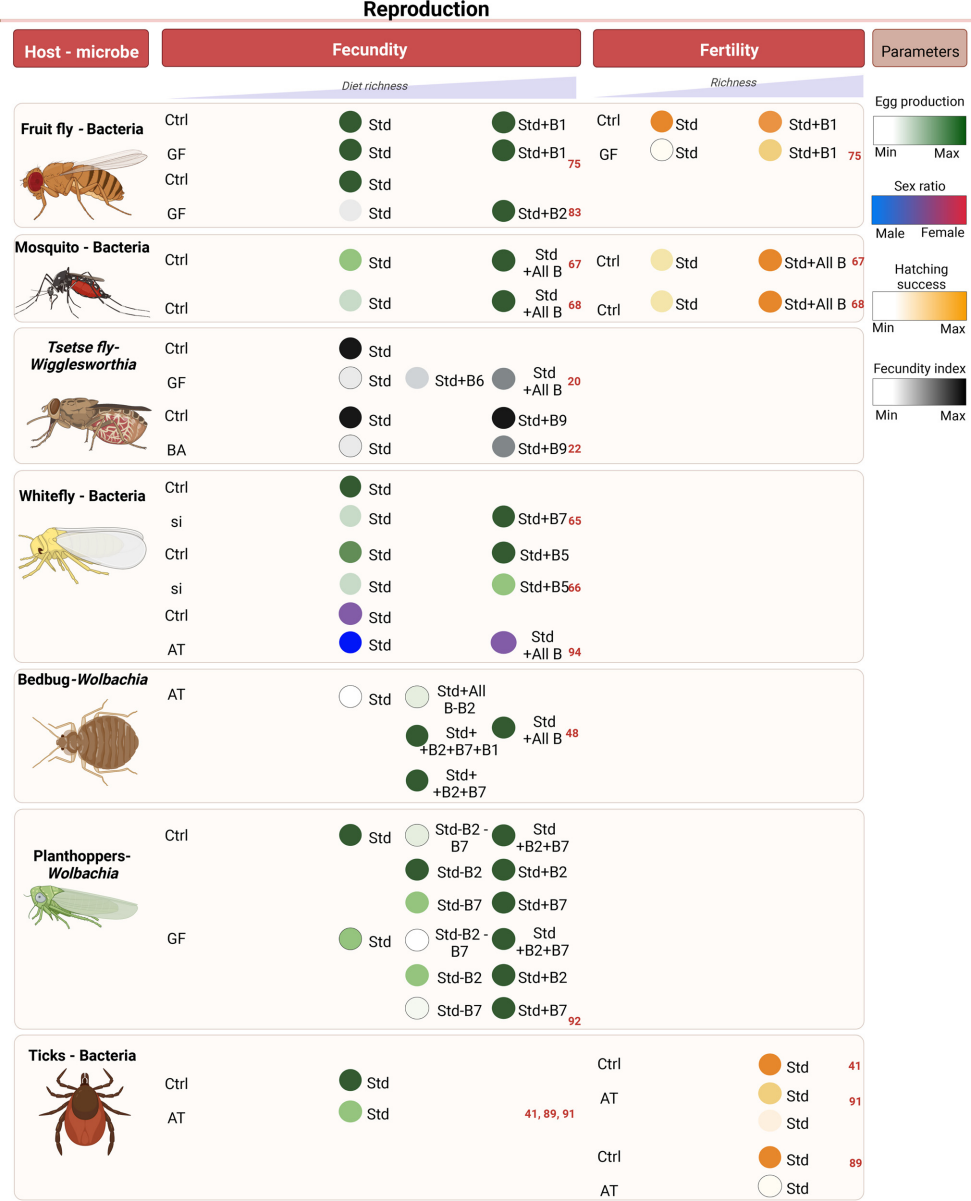
Influence of B vitamins on insect reproduction (20, 22, 41, 48, 65–68, 75, 83, 89, 91, 92, 94). AT, antibiotic treated; GF, germfree; Ctrl, microbiota control (which can either mean conventionally reared or gnotobiotic, with AT and GF, respectively); Si, gene silencing by double-strand RNA injection; BA, blocking agent (antagonist) used; Std, standard diet, neither impoverished nor enriched (a holidic diet is considered standard when it contains all the requirements for normal physiology). The image was created with BioRender.

B vitamin requirements for insect reproduction was first studied via diet manipulation and antivitamin treatments. Several antivitamins were notably reported to inhibit egg production by *Musca viscinis*, an effect that could partly be rescued using the corresponding vitamin (86). Later, Saxena and Kaul were surprised to see how little effect vitamin deficiency in the diet had on *Oryzaephilus mercator* (*Coleoptera*) fertility and discussed that this may be due to microbe-dependent production of vitamins (87). More recent studies on mosquitoes and bedbugs also pointed to a positive effect of sugar diet supplementation with B vitamins on male and female fertility. These dietary supplements specifically increased male fertilization capacity during forced-mating experiments, egg production by females, egg hatching success, and/or viability of the hatched larvae (40, 67, 68). This corroborates reports of negative effects on female fecundity and/or fertility found in *Anopheles* and *Aedes* upon antibiotic treatment, effects that are rescued by *Serratia* or Enterobacter colonization in *Anopheles* (71, 88). In *Aedes*, the microbiota promotes fecundity via hemolysis in the blood bolus, while colonization with E. coli HS, a nonhemolytic bacterial strain, does not affect fertility (Fig. 4) (61, 71). While the link has not been experimentally established, these observations would fit with a model where B vitamins are released by bacteria via promoting hemolysis, hence fostering mosquito fertility. Alternatively, some bacteria such as *Serratia* and Enterobacter may produce a higher or more constant amounts of B vitamins than E. coli. In ticks, maternally inherited endosymbionts are essential for oviposition (lone star tick) and egg development (lone star tick and *Riphicephalus*), but the involvement of B vitamins has not been specifically investigated so far (Fig. 4) (41, 89–91).

When focusing on specific vitamins, there is evidence that riboflavin and biotin particularly affect insect fecundity (Fig. 4). The addition of dietary riboflavin improves egg production in conventionally reared fruit flies (76). While oogenesis is reduced in germfree flies as a result of decreased ATP levels, riboflavin supplementation is sufficient to reverse the effect (83). Riboflavin supplementation has also been found to restore fecundity in bedbugs cured from *Wolbachia* via antibiotic treatment (48). A biotin dietary supplement also promotes fecundity in *Wolbachia-*cured bedbugs (49). Genomic analyses showed that the riboflavin biosynthesis pathway is often complete in *Wolbachia*, while the biotin biosynthesis pathway is complete in some *Cimex* and *Paracimex* bedbug species, as well as in some planthoppers and missing in the other insect genera tested (Fig. 2) (43, 48, 92). A similar situation occurs in planthoppers, where the presence of *Wolbachia* enhances egg production. Experiments based on the depletion or addition of dietary biotin and/or riboflavin indicate that this impact is driven by the provision of these vitamins by *Wolbachia*, the role of biotin being particularly important (92). In whiteflies as well, the removal of symbionts *Portiera* and *Hamiltonella* leads to reduced titers of pantothenate and biotin, respectively, and scant egg production (65, 66). Silencing host genes of the pantothenate and biotin biosynthesis pathways (inherited from bacteria by horizontal gene transfer, as mentioned in the previous section) recapitulates antibiotic treatments against *Portiera* and *Hamiltonella*, respectively. Again, this indicates that whiteflies and their symbionts collaborate to support the adult host requirement on B vitamins.

On the contrary, thiamine deficiency does not appear to affect egg production in germfree *Drosophila* (Fig. 4). When fed with a thiamine-deprived diet, F_1_ larvae hatched from those eggs can fulfill development if they are conventionally reared but not if they are germfree (75). The latter observation points back to an impact of microbial thiamine in development rather than reproduction, as mentioned above (4).

Tsetse flies are adenotrophic viviparous, their larvae are carried and fed with milk within the female uterus and get born in a prepupal stage. They harbor an obligatory symbiont, *Wigglesworthia*, so no cured line can be established over generations, yet it is possible to get a *Wigglesworthia*-cured progeny of “aposymbionts” after treating a female with antibiotics. These aposymbionts are reproductively sterile and do not produce folate. To study the role of folate in tsetse flies, Snyder and Rio treated conventionally reared flies with glyphosate, which inhibits the biosynthesis of chorismate, a precursor of folate (22). Since this pathway is present in *Wigglesworthia* but absent in the fly genome (Fig. 2), this treatment specifically targets folate production by the symbiont. This treatment leads to a delay in blood meal digestion, a reduction in the intrauterine area and a delay in time to pupation, which can be partially rescued by folate supplementation (22). Proline is a cornerstone of tsetse fly metabolism during lactation and its biosynthesis depends on alanine-glyoxylate transaminase, a protein which requires pyridoxine as a cofactor. Experiments based on antibiotic treatments and vitamin supplementation have shown that *Wigglesworthia* pyridoxine is essential for lactation (20, 93).

Besides such “typical” effects of vitamins to support metabolism and egg production, microbe-derived B vitamins have also been suggested to impact other aspects of reproduction, affecting sex ratio and compatibility between mating partners. First, the removal of whitefly symbionts has been found to affect fertilization process. Antibiotic treatments targeting *Hamiltonella* or *Arsenophonous* lead to an imbalance in the sex ratio of the progeny, reducing the proportion of females from 60% to 20%. This is due to a loss in fertilization leading to arrhenotokous (i.e., male-producing) parthenogenesis, which can be rescued using a cocktail of B vitamins (94). Thus, these symbionts increase the proportion of females in the progeny by avoiding a male-producing parthenogenesis, which would be detrimental for their vertical transmission. Interestingly, some other endosymbionts have been found to promote thelytokous (i.e., female-producing) parthenogenesis (albeit not reportedly via B vitamin related mechanisms). Whether by avoiding arrhenotokous parthenogenesis or by inducing thelytokous parthenogenesis, in both cases they are increasing their chance of vertical transmission (95). *Wolbachia* is also known to manipulate reproduction via cytoplasmic incompatibility; colonized males are infertile unless they mate with a female colonized with the same strain of *Wolbachia*. This incompatibility promotes *Wolbachia* colonization in a population as it favors colonized females, which can be fertilized by any male and will vertically transmit the endosymbiont. A recent comparison of *Wolbachia* genomes from 500 host species, including nematodes and arthropods, showed a correlation between the presence of a riboflavin transporter in *Wolbachia* genome and cytoplasmic incompatibility (45), yet functional links have not yet been characterized.

## CONCLUSION

In sum, the microbiota, including symbionts and transient microbes, strongly affects the physiology of its host throughout the reproductive cycle via the provision of B vitamins. While it is convenient to classify microbes as primary, secondary symbionts or reproductive manipulators for instance, examples treated in this review highlight that one microbe may be differently classified depending on the conditions. Host-microbe interactions can be placed on a gradient of parasitism/mutualism rather than in strict classes. For instance, while *Wolbachia* is generally a reproductive manipulator in insects and a mutualist in nematodes (96, 97), some lines of evidence point to its ability to provide B vitamins to some insects (40, 44, 48, 54, 92). Such infections, favored by a combination between a pressure (here, reproductive manipulation) and a slight positive outcome of colonization on the host, are termed “Jekyll and Hyde” infections (48). It remains unclear whether they also occur in a wider range of insect species, where the positive fitness component may be undetected due to redundancy with nutrient provision by other members of the microbiota.

In immature stages, microbe-derived B vitamins are important for completion of development and survival. Depending on the associations, microbes provide B vitamins that are absent in the host diet or complement diet provision, ensuring development in scarce dietary conditions. In adults, microbe-derived B vitamins tend to prolong the life span, affect vector competence and participate in reproductive success via egg production, fertilization, and lactation. Interestingly, it seems that some vitamins are nonspecifically cited in studies on development, life span, or reproduction, while some others appear more often in studies on larvae or adults. For instance, folate is implicated in the development of *Drosophila*, tsetse flies, mosquitoes, bedbugs, and aphids but is reportedly not so critical in adult life span studies. Biotin is important for life span and/or egg production in adult *Drosophila*, whiteflies, bedbugs, and planthoppers. Riboflavin is found to be important during development, as well as during adulthood in dipterans, bedbugs, and aphids. These observations may be biased by the fact that most diets include unknown concentrations of different vitamins. This issue could be addressed using chemically defined diets in germfree and gnotobiotic hosts in order to clearly define the contribution of microbes to their host’s physiology.

While a number of requirements on vitamins for specific phenotypic readouts have been reported, the underlying mechanisms have rarely been uncovered. An exception to this rule is the demonstration of the role of pyridoxine in tsetse flies as a specific cofactor of *agat* for milk production. Given that vitamins have conserved roles in eukaryotic cells, a full characterization of their mechanisms in insects is central to developing a better understanding the role of the microbiota in animals, which may have a wide spectrum of applications.

## References

[1] Douglas AE. 2017. The B vitamin nutrition of insects: the contributions of diet, microbiome and horizontally acquired genes. Curr Opin Insect Sci 23:65–69. doi:10.1016/j.cois.2017.07.012. 29129284

[2] Kucuk RA. 2020. Gut bacteria in the holometabola: a review of obligate and facultative symbionts. J Insect Sci 20. doi:10.1093/jisesa/ieaa084. PMC7433766 32809024

[3] Yoshii K, Hosomi K, Sawane K, Kunisawa J. 2019. Metabolism of dietary and microbial vitamin B family in the regulation of host immunity. Front Nutr 6:48. doi:10.3389/fnut.2019.00048. 31058161 PMC6478888

[4] Consuegra J, Grenier T, Baa-Puyoulet P, Rahioui I, Akherraz H, Gervais H, Parisot N, da Silva P, Charles H, Calevro F, Leulier F. 2020. *Drosophila*-associated bacteria differentially shape the nutritional requirements of their host during juvenile growth. PLoS Biol 18:e3000681. doi:10.1371/journal.pbio.3000681. 32196485 PMC7112240

[5] Engel P, Moran NA. 2013. The gut microbiota of insects: diversity in structure and function. FEMS Microbiol Rev 37:699–735. doi:10.1111/1574-6976.12025. 23692388

[6] Shin SC, Kim SH, You H, Kim B, Kim AC, Lee KA, Yoon JH, Ryu JH, Lee WJ. 2011. *Drosophila* microbiome modulates host developmental and metabolic homeostasis via insulin signaling. Science 334:670–674. doi:10.1126/science.1212782. 22053049

[7] Storelli G, Defaye A, Erkosar B, Hols P, Royet J, Leulier F. 2011. *Lactobacillus plantarum* promotes drosophila systemic growth by modulating hormonal signals through TOR-dependent nutrient sensing. Cell Metab 14:403–414. doi:10.1016/j.cmet.2011.07.012. 21907145

[8] Blewett M, Fraenkel G. 1944. Intracellular symbiosis and vitamin requirements of two insects, *Lasioderma serricorne* and *Sitodrepa panicea*. Proc R Soc Lond B Biol Sci 132:212–221.

[9] Brecher G, Wigglesworth VB. 1944. The transmission of *Actinomyces rhodnii* Erikson in *Rhodnius prolixus* Stål (hemiptera) and its influence on the growth of the host. Parasitology 35:220–224. doi:10.1017/S0031182000021648.

[10] Dadd RH. 1973. Insect nutrition: current developments and metabolic implications. Annu Rev Entomol 18:381–420. doi:10.1146/annurev.en.18.010173.002121. 4218469

[11] Correa MA, Matusovsky B, Brackney DE, Steven B. 2018. Generation of axenic *Aedes aegypti* demonstrate live bacteria are not required for mosquito development. Nat Commun 9:4464. doi:10.1038/s41467-018-07014-2. 30367055 PMC6203775

[12] Piper MDW, Blanc E, Leitão-Gonçalves R, Yang M, He X, Linford NJ, Hoddinott MP, Hopfen C, Soultoukis GA, Niemeyer C, Kerr F, Pletcher SD, Ribeiro C, Partridge L. 2014. A holidic medium for *Drosophila melanogaster*. Nat Methods 11:100–105. doi:10.1038/nmeth.2731. 24240321 PMC3877687

[13] Biedermann PHW, Vega FE. 2020. Ecology and evolution of insect-fungus mutualisms. Annu Rev Entomol 65:431–455. doi:10.1146/annurev-ento-011019-024910. 31610133

[14] Gurung K, Wertheim B, Falcao Salles J. 2019. The microbiome of pest insects: it is not just bacteria. Entomol Exp Appl 167:156–170. doi:10.1111/eea.12768.

[15] Voronovsky AY, Abbas CA, Dmytruk K, Ishchuk OP, Kshanovska B.v, Sybirna KA, Gaillardin C, Sibirny AA. 2004. *Candida famata* (*Debaryomyces hansenii*) DNA sequences containing genes involved in riboflavin synthesis. Yeast 21:1307–1316. doi:10.1002/yea.1182. 15543522

[16] Hart RJ, Lawres L, Fritzen E, Ben Mamoun C, Aly ASI. 2014. *Plasmodium yoelii* vitamin B_5_ pantothenate transporter candidate is essential for parasite transmission to the mosquito. Sci Rep 4:5665. doi:10.1038/srep05665. 25012929 PMC4092334

[17] Onchuru TO, Martinez AJ, Kaltenpoth M. 2018. The cotton stainer’s gut microbiota suppresses infection of a cotransmitted trypanosomatid parasite. Mol Ecol 27:3408–3419. doi:10.1111/mec.14788. 29972876

[18] Rio RVM, Jozwick AKS, Savage AF, Sabet A, Vigneron A, Wu Y, Aksoy S, Weiss BL. 2019. Mutualist-provisioned resources impact vector competency. mBio 10:e00018-19. doi:10.1128/mBio.00018-19. 31164458 PMC6550517

[19] Herren JK, Paredes JC, Schüpfer F, Arafah K, Bulet P, Lemaitre B. 2014. Insect endosymbiont proliferation is limited by lipid availability. Elife 3:e02964. doi:10.7554/eLife.02964. 25027439 PMC4123717

[20] Michalkova V, Benoit JB, Weiss BL, Attardo GM, Aksoy S. 2014. Vitamin B_6_ generated by obligate symbionts is critical for maintaining proline homeostasis and fecundity in tsetse flies. Appl Environ Microbiol 80:5844–5853. doi:10.1128/AEM.01150-14. 25038091 PMC4178588

[21] Nakabachi A, Ishikawa H. 1999. Provision of riboflavin to the host aphid, *Acyrthosiphon pisum*, by endosymbiotic bacteria, *Buchnera*. J Insect Physiol 45:1–6. doi:10.1016/S0022-1910(98)00104-8. 12770389

[22] Snyder AK, Rio RVM. 2015. “*Wigglesworthia morsitans*” folate (vitamin B_9_) biosynthesis contributes to tsetse host fitness. Appl Environ Microbiol 81:5375–5386. doi:10.1128/AEM.00553-15. 26025907 PMC4510189

[23] Husnik F, McCutcheon JP. 2018. Functional horizontal gene transfer from bacteria to eukaryotes. Nat Rev Microbiol 16:67–79. doi:10.1038/nrmicro.2017.137. 29176581

[24] McCutcheon JP, Moran NA. 2011. Extreme genome reduction in symbiotic bacteria. Nat Rev Microbiol 10:13–26. doi:10.1038/nrmicro2670. 22064560

[25] Felsenstein J. 1974. The evolutionary advantage of recombination. Genetics 78:737–756. doi:10.1093/genetics/78.2.737. 4448362 PMC1213231

[26] Morris JJ, Lenski RE, Zinser ER. 2012. The Black Queen Hypothesis: evolution of dependencies through adaptive gene loss. mBio 3:e00036-12. doi:10.1128/mBio.00036-12. 22448042 PMC3315703

[27] Gupta A, Nair S. 2020. Dynamics of insect–microbiome interaction influence host and microbial symbiont. Front Microbiol 11. 10.3389/fmicb.2020.01357 PMC7333248 32676060

[28] Putnam EE, Goodman AL. 2020. B vitamin acquisition by gut commensal bacteria. PLoS Pathog 16:e1008208. doi:10.1371/journal.ppat.1008208. 31971969 PMC6977713

[29] Coon KL, Vogel KJ, Brown MR, Strand MR. 2014. Mosquitoes rely on their gut microbiota for development. Mol Ecol 23:2727–2739. doi:10.1111/mec.12771. 24766707 PMC4083365

[30] Hegde S, Voronin D, Casas-Sanchez A, Saldaña MA, Heinz E, Acosta-Serrano A, Popov VL, Chopra AK, Hughes LG. 2019. Gut-associated bacteria invade the midgut epithelium of *Aedes aegypti* and stimulate innate immunity and suppress Zika virus infection in cells. bioRxiv. https://www.biorxiv.org/content/10.1101/866897v2.

[31] Duron O, Gottlieb Y. 2020. Convergence of nutritional symbioses in obligate blood feeders. Trends Parasitol 36:816–825. doi:10.1016/j.pt.2020.07.007. 32811753

[32] Acevedo-Rocha CG, Gronenberg LS, Mack M, Commichau FM, Genee HJ. 2019. Microbial cell factories for the sustainable manufacturing of B vitamins. Curr Opin Biotechnol 56:18–29. doi:10.1016/j.copbio.2018.07.006. 30138794

[33] Kanehisa M, Furumichi M, Sato Y, Ishiguro-Watanabe M, Tanabe M. 2021. KEGG: integrating viruses and cellular organisms. Nucleic Acids Res 49:D545–D551. doi:10.1093/nar/gkaa970. 33125081 PMC7779016

[34] Roje S. 2007. Vitamin B biosynthesis in plants. Phytochemistry 68:1904–1921. doi:10.1016/j.phytochem.2007.03.038. 17512961

[35] Bakula M. 1969. The persistence of a microbial flora during postembryogenesis of *Drosophila melanogaster*. J Invertebr Pathol 14:365–374. doi:10.1016/0022-2011(69)90163-3. 4904970

[36] Baines BYS. 1956. The role of symbiotic bacteria in the nutrition of *Rhodnius prolixus* (Hemiptera). Exp Biol 3.

[37] Ben-Yakir D. 1987. Growth retardation of *Rhodnius prolixus* symbionts by immunizing host against *Nocardia* (*Rhodococcus*) *rhodnii*. J Insect Physiol 33:379–383. doi:10.1016/0022-1910(87)90015-1.

[38] Tobias NJ, Eberhard FE, Guarneri AA. 2020. Enzymatic biosynthesis of B-complex vitamins is supplied by diverse microbiota in the *Rhodnius prolixus* anterior midgut following *Trypanosoma cruzi* infection. Comput Struct Biotechnol J 18:3395–3401. doi:10.1016/j.csbj.2020.10.031. 33294135 PMC7691439

[39] Duron O, Binetruy F, Noël V, Cremaschi J, McCoy KD, Arnathau C, Plantard O, Goolsby J, Pérez de León AA, Heylen DJAA, van Oosten AR, Gottlieb Y, Baneth G, Guglielmone AA, Estrada-Peña A, Opara MN, Zenner L, Vavre F, Chevillon C. 2017. Evolutionary changes in symbiont community structure in ticks. Mol Ecol 26:2905–2921. doi:10.1111/mec.14094. 28281305

[40] Hosokawa T, Koga R, Kikuchi Y, Meng XY, Fukatsu T. 2010. *Wolbachia* as a bacteriocyte-associated nutritional mutualist. Proc Natl Acad Sci USA 107:769–774. doi:10.1073/pnas.0911476107. 20080750 PMC2818902

[41] Zhong J, Jasinskas A, Barbour AG. 2007. Antibiotic treatment of the tick vector *Amblyomma americanum* reduced reproductive fitness. PLoS One 2:e405. doi:10.1371/journal.pone.0000405. 17476327 PMC1852332

[42] Salcedo-Porras N, Umaña-Diaz C, de Oliveira Barbosa Bitencourt R, Lowenberger C. 2020. The role of bacterial symbionts in triatomines: an evolutionary perspective. Microorganisms 8:1438–25. doi:10.3390/microorganisms8091438. 32961808 PMC7565714

[43] Balvín O, Roth S, Talbot B, Reinhardt K. 2018. Co-speciation in bedbug *Wolbachia* parallel the pattern in nematode hosts. Sci Rep 8:8797. doi:10.1038/s41598-018-25545-y. 29891919 PMC5995804

[44] Gerth M, Bleidorn C. 2016. Comparative genomics provides a timeframe for *Wolbachia* evolution and exposes a recent biotin synthesis operon transfer. Nat Microbiol 2:16241. doi:10.1038/nmicrobiol.2016.241. 28005061

[45] Scholz M, Albanese D, Tuohy K, Donati C, Segata N, Rota-Stabelli O. 2020. Large-scale genome reconstructions illuminate *Wolbachia* evolution. Nat Commun 11. doi:10.1038/s41467-020-19016-0. PMC7568565 33067437

[46] Ishikawa H. 1982. Host-symbiont interactions in the protein synthesis in the pea aphid, *Acyrthosiphon pisum*. Insect Biochem 12:613–622. doi:10.1016/0020-1790(82)90048-8.

[47] Blow F, Bueno E, Clark N, Zhu DT, Chung SH, Güllert S, Schmitz RA, Douglas AE. 2020. B-vitamin nutrition in the pea aphid-*Buchnera* symbiosis. J Insect Physiol 126:104092. doi:10.1016/j.jinsphys.2020.104092. 32763248

[48] Moriyama M, Nikoh N, Hosokawa T, Fukatsu T. 2015. Riboflavin provisioning underlies *Wolbachia*’s fitness contribution to its insect host. mBio 6:e01732-15. doi:10.1128/mBio.01732-15. 26556278 PMC4659472

[49] Nikoh N, Hosokawa T, Moriyama M, Oshima K, Hattori M, Fukatsu T. 2014. Evolutionary origin of insect-*Wolbachia* nutritional mutualism. Proc Natl Acad Sci USA 111:10257–10262. doi:10.1073/pnas.1409284111. 24982177 PMC4104916

[50] Romanov DA, Zakharov IA, Shaikevich EV. 2020. *Wolbachia*, *Spiroplasma*, and *Rickettsia* symbiotic bacteria in aphids (Aphidoidea). Vavilovskii Zhurnal Genet Selektsii 24:673–682. doi:10.18699/VJ20.661. 33659853 PMC7716544

[51] Kaur R, Shropshire JD, Cross KL, Leigh B, Mansueto AJ, Stewart V, Bordenstein SR, Bordenstein SR. 2021. Living in the endosymbiotic world of *Wolbachia*: a centennial review. Cell Host Microbe 29:879–893. doi:10.1016/j.chom.2021.03.006. 33945798 PMC8192442

[52] Fan HW, Lu JB, Ye YX, Yu XP, Zhang CX. 2016. Characteristics of the draft genome of “*Candidatus* Arsenophonus nilaparvatae,” a facultative endosymbiont of *Nilaparvata lugens*. Insect Sci 23:478–486. doi:10.1111/1744-7917.12318. 26792263

[53] Karimi S, Askari Seyahooei M, Izadi H, Bagheri A, Khodaygan P. 2019. Effect of arsenophonus endosymbiont elimination on fitness of the date palm hopper, *Ommatissus lybicus* (Hemiptera: Tropiduchidae). Environ Entomol 48:614–622. doi:10.1093/ee/nvz047. 31095275

[54] Qu L-Y, Lou Y-H, Fan H-W, Ye Y-X, Huang H-J, Hu M-Q, Zhu Y-N, Zhang C-X. 2013. Two endosymbiotic bacteria, *Wolbachia* and *Arsenophonus*, in the brown planthopper *Nilaparvata lugens*. Symbiosis 61:47–53. doi:10.1007/s13199-013-0256-9.

[55] Xue J, Zhou X, Zhang CX, Yu LL, Fan HW, Wang Z, Xu HJ, Xi Y, Zhu ZR, Zhou WW, Pan PL, Li BL, Colbourne JK, Noda H, Suetsugu Y, Kobayashi T, Zheng Y, Liu S, Zhang R, Liu Y, Luo YD, Fang DM, Chen Y, Zhan DL, Lv XD, Cai Y, Wang ZB, Huang HJ, Cheng RL, Zhang XC, Lou YH, Yu B, Zhuo JC, Ye YX, Zhang WQ, Shen ZC, Yang HM, Wang J, Wang J, Bao YY, Cheng JA. 2014. Genomes of the rice pest brown planthopper and its endosymbionts reveal complex complementary contributions for host adaptation. Genome Biol 15:521. doi:10.1186/s13059-014-0521-0. 25609551 PMC4269174

[56] Sazama EJ, Bosch MJ, Shouldis CS, Ouellette SP, Wesner JS. 2017. Incidence of *Wolbachia* in aquatic insects. Ecol Evol 7:1165–1169. doi:10.1002/ece3.2742. 28303186 PMC5306009

[57] Blatch S, Meyer KW, Harrison JF. 2010. Effects of dietary folic acid level and symbiotic folate production on fitness and development in the fruit fly *Drosophila melanogaster*. Fly (Austin) 4:312–319. doi:10.4161/fly.4.4.13258. 20855945

[58] Blatch S, Stabler SP, Harrison JF. 2015. The effects of folate intake on DNA and single-carbon pathway metabolism in the fruit fly *Drosophila melanogaster* compared to mammals. Comp Biochem Physiol B Biochem Mol Biol 189:34–39. doi:10.1016/j.cbpb.2015.07.007. 26219578

[59] Wong AC-N, Dobson AJ, Douglas AE. 2014. Gut microbiota dictates the metabolic response of *Drosophila* to diet. J Exp Biol 217:1894–1901. doi:10.1242/jeb.101725. 24577449 PMC4037322

[60] Consuegra J, Grenier T, Akherraz H, Rahioui I, Gervais H, da Silva P, Leulier F. 2020. Metabolic cooperation among commensal bacteria supports *Drosophila* juvenile growth under nutritional stress. iScience 23:101232. doi:10.1016/j.isci.2020.101232. 32563155 PMC7305377

[61] Romoli O, Schönbeck JC, Hapfelmeier S, Gendrin M. 2021. Production of germ-free mosquitoes via transient colonization allows stage-specific investigation of host–microbiota interactions. Nat Commun 12:942. doi:10.1038/s41467-021-21195-3. 33574256 PMC7878806

[62] Wang Y, Eum JH, Harrison RE, Valzania L, Yang X, Johnson JA, Huck DT, Brown MR, Strand MR. 2021. Riboflavin instability is a key factor underlying the requirement of a gut microbiota for mosquito development. Proc Natl Acad Sci USA 118:e2101080118. doi:10.1073/pnas.2101080118. 33827929 PMC8053949

[63] Buchon N, Broderick NA, Chakrabarti S, Lemaitre B. 2009. Invasive and indigenous microbiota impact intestinal stem cell activity through multiple pathways in *Drosophila*. Genes Dev 23:2333–2344. doi:10.1101/gad.1827009. 19797770 PMC2758745

[64] McCutcheon JP, Boyd BM, Dale C. 2019. The life of an insect endosymbiont from the cradle to the grave. Curr Biol 29:R485–R495. doi:10.1016/j.cub.2019.03.032. 31163163

[65] Ren F-R, Sun X, Wang T-Y, Yao Y-L, Huang Y-Z, Zhang X, Luan J-B. 2020. Biotin provisioning by horizontally transferred genes from bacteria confers animal fitness benefits. ISME J 14:2542–2553. doi:10.1038/s41396-020-0704-5. 32572143 PMC7490365

[66] Ren F-R, Sun X, Wang T, Yan J-Y, Yao Y-L, Li C-Q, Luan J-B. 2021. Pantothenate mediates the coordination of whitefly and symbiont fitness. ISME J 15:1655–1667. doi:10.1038/s41396-020-00877-8. 33432136 PMC8163847

[67] Phasomkusolsil S, Pantuwatana K, Tawong J, Khongtak W, Kertmanee Y, Monkanna N, Khaosanorh S, Wanja EW, Davidson SA. 2017. Sugar and multivitamin diet effects on the longevity and mating capacity of laboratory-reared male anopheline mosquitoes. J Am Mosq Control Assoc 33:175–183. doi:10.2987/17-6634R.1. 28854115

[68] Tan SB, Nazni WA, Misni S, Zuraini Z, Lee HL. 2016. Effects of vitamin B fortified sucrose solution on the longevity and reproductive potentials of laboratory-bred *Culex quinquefasciatus* say adult. Trop Biomed 33:141–148. 33579151

[69] Giraud É, Varet H, Legendre R, Sismeiro O, Aubry F, Dabo S, Dickson LB, Valiente Moro C, Lambrechts L. 2022. Mosquito-bacterium interactions during larval development trigger metabolic changes with carry-over effects on adult fitness. Mol Ecol 31:1444–1460. doi:10.1111/mec.16327. 34905257

[70] Rodgers FH, Gendrin M, Wyer CAS, Christophides GK. 2017. Microbiota-induced peritrophic matrix regulates midgut homeostasis and prevents systemic infection of malaria vector mosquitoes. PLoS Pathog 13:e1006391. doi:10.1371/journal.ppat.1006391. 28545061 PMC5448818

[71] Gaio ADO, Gusmão DS, Santos A.v, Berbert-Molina MA, Pimenta PFP, Lemos FJA. 2011. Contribution of midgut bacteria to blood digestion and egg production in *Aedes aegypt*i (diptera: Culicidae) (L.). Parasit Vectors 4:105. doi:10.1186/1756-3305-4-105. 21672186 PMC3125380

[72] Hyde J, Correa MA, Hughes GL, Steven B, Brackney DE. 2020. Limited influence of the microbiome on the transcriptional profile of female *Aedes aegypti* mosquitoes. Sci Rep 10:10880. doi:10.1038/s41598-020-67811-y. 32616765 PMC7331810

[73] Landenberger A, Kabil H, Harshman LG, Zempleni J. 2004. Biotin deficiency decreases life span and fertility but increases stress resistance in *Drosophila melanogaster*. J Nutr Biochem 15:591–600. doi:10.1016/j.jnutbio.2004.04.006. 15542350

[74] Neophytou C, Pitsouli C. 2022. Biotin controls intestinal stem cell mitosis and host-microbiome interactions. Cell Rep 38:110505. doi:10.1016/j.celrep.2022.110505. 35263602

[75] Sannino DR, Dobson AJ, Edwards K, Angert ER, Buchon N. 2018. The *Drosophila melanogaster* gut microbiota provisions thiamine to its host. mBio 9:e00155-18. doi:10.1128/mBio.00155-18. 29511074 PMC5845000

[76] Zou Y-X, Ruan M-H, Luan J, Feng X, Chen S, Chu Z-Y. 2017. Anti-aging effect of riboflavin via endogenous antioxidant in fruit fly *Drosophila melanogaster*. J Nutr Health Aging 21:314–319. doi:10.1007/s12603-016-0752-8.28244572

[77] Duron O, Schneppat UE, Berthomieu A, Goodman SM, Droz B, Paupy C, Obame Nkoghe J, Rahola N, Tortosa P. 2014. Origin, acquisition and diversification of heritable bacterial endosymbionts in louse flies and bat flies. Mol Ecol 23:2105–2117. doi:10.1111/mec.12704. 24612422

[78] Hosokawa T, Nikoh N, Koga R, Satô M, Tanahashi M, Meng X-Y, Fukatsu T. 2012. Reductive genome evolution, host–symbiont co-speciation and uterine transmission of endosymbiotic bacteria in bat flies. ISME J 6:577–587. doi:10.1038/ismej.2011.125. 21938025 PMC3280136

[79] Šochová E, Husník F, Nováková E, Halajian A, Hypša V. 2017. Arsenophonus and Sodalis replacements shape evolution of symbiosis in louse flies. PeerJ 5:e4099. doi:10.7717/peerj.4099. 29250466 PMC5729840

[80] Cheng D, Chen S, Huang Y, Pierce NE, Riegler M, Yang F, Zeng L, Lu Y, Liang G, Xu Y. 2019. Symbiotic microbiota may reflect host adaptation by resident to invasive ant species. PLoS Pathog 15:e1007942. doi:10.1371/journal.ppat.1007942. 31323076 PMC6668852

[81] Kirkness EF, Haas BJ, Sun W, Braig HR, Perotti MA, Clark JM, Lee SH, Robertson HM, Kennedy RC, Elhaik E, Gerlach D, Kriventseva E, Elsik CG, Graur D, Hill CA, Veenstra JA, Walenz B, Tubio JMC, Ribeiro JMC, Rozas J, Johnston JS, Reese JT, Popadic A, Tojo M, Raoult D, et al. 2010. Genome sequences of the human body louse and its primary endosymbiont provide insights into the permanent parasitic lifestyle. Proc Natl Acad Sci USA 107:12168–12173. doi:10.1073/pnas.1003379107. 20566863 PMC2901460

[82] Hart RJ, Cornillot E, Abraham A, Molina E, Nation CS, Ben Mamoun C, Aly ASI. 2016. Genetic characterization of plasmodium putative pantothenate kinase genes reveals their essential role in malaria parasite transmission to the mosquito. Sci Rep 6:1–10. doi:10.1038/srep33518. 27644319 PMC5028760

[83] Gnainsky Y, Zfanya N, Elgart M, Omri E, Brandis A, Mehlman T, Itkin M, Malitsky S, Adamski J, Soen Y. 2021. Systemic regulation of host energy and oogenesis by microbiome-derived mitochondrial coenzymes. Cell Rep 34:108583. doi:10.1016/j.celrep.2020.108583. 33406416

[84] Mao M, Bennett GM. 2020. Symbiont replacements reset the co-evolutionary relationship between insects and their heritable bacteria. ISME J 14:1384–1395. doi:10.1038/s41396-020-0616-4. 32076126 PMC7242365

[85] Wu Z, Yang L, He Q, Zhou S. 2020. Regulatory mechanisms of vitellogenesis in insects. Front Cell Dev Biol 8:593613. doi:10.3389/fcell.2020.593613. 33634094 PMC7901893

[86] Bergmann ED, Rabinovitz M, Levinson ZH. 1959. The synthesis and biological availability of some lower homologs of cholesterol. J Am Chem Soc 81:1239–1243. doi:10.1021/ja01514a056.

[87] Saxena SC, Kaul S. 1974. Qualitative vitamin requirements of *Oryzaephilus mercator* Fauvel and their deficiency effects on the survival and growth of F_1_ progeny 49-54. doi:10.3109/13813457409070451. 4137150

[88] Ezemuoka LC, Akorli EA, Aboagye-Antwi F, Akorli J. 2020. Mosquito midgut *Enterobacter cloacae* and *Serratia marcescens* affect the fitness of adult female *Anopheles gambiae* s.l. PLoS One 15:e0238931. doi:10.1371/journal.pone.0238931. 32946471 PMC7500640

[89] Guizzo MG, Parizi LF, Nunes RD, Schama R, Albano RM, Tirloni L, Oldiges DP, Vieira RP, Oliveira WHC, Leite MDS, Gonzales SA, Farber M, Martins O, Vaz IDS, Oliveira PL. 2017. A *Coxiella* mutualist symbiont is essential to the development of *Rhipicephalus microplus*. Sci Rep 7. doi:10.1038/s41598-017-17309-x. PMC5730597 29242567

[90] Hunter DJ, Torkelson JL, Bodnar J, Mortazavi B, Laurent T, Deason J, Thephavongsa K, Zhong J. 2015. The *Rickettsia* endosymbiont of *Ixodes pacificus* contains all the genes of *de novo* folate biosynthesis. PLoS One 10:e0144552. doi:10.1371/journal.pone.0144552. 26650541 PMC4674097

[91] Li L-H, Zhang Y, Zhu D. 2018. Effects of antibiotic treatment on the fecundity of *Rhipicephalus haemaphysaloides* ticks. Parasit Vectors 11:242. doi:10.1186/s13071-018-2807-7. 29653599 PMC5899350

[92] Ju JF, Bing XL, Zhao DS, Guo Y, Xi Z, Hoffmann AA, Zhang KJ, Huang HJ, Gong JT, Zhang X, Hong XY. 2020. *Wolbachia* supplement biotin and riboflavin to enhance reproduction in planthoppers. ISME J 14:676–687. doi:10.1038/s41396-019-0559-9. 31767943 PMC7031331

[93] Snyder AK, Deberry JW, Runyen-Janecky L, Rio RVM. 2010. Nutrient provisioning facilitates homeostasis between tsetse fly (Diptera: Glossinidae) symbionts. Proc Biol Sci 277:2389–2397. doi:10.1098/rspb.2010.0364. 20356887 PMC2894912

[94] bin Wang Y, Ren FR, Yao YL, Sun X, Walling LL, Li NN, Bai B, Bao XY, Xu XR, Luan JB. 2020. Intracellular symbionts drive sex ratio in the whitefly by facilitating fertilization and provisioning of B vitamins. ISME J 14:2923–2935. doi:10.1038/s41396-020-0717-0. 32690936 PMC7784916

[95] Massey JH, Newton ILG. 2022. Diversity and function of arthropod endosymbiont toxins. Trends Microbiol 30:185–198. doi:10.1016/j.tim.2021.06.008. 34253453 PMC8742837

[96] Brown AMV, Wasala SK, Howe DK, Peetz AB, Zasada IA, Denver DR. 2018. Comparative genomics of *Wolbachia*-*Cardinium* dual endosymbiosis in a plant-parasitic nematode. Front Microbiol 9:2482. doi:10.3389/fmicb.2018.02482. 30459726 PMC6232779

[97] Li Z, Carlow CKS. 2012. Characterization of transcription factors that regulate the type IV secretion system and riboflavin biosynthesis in *Wolbachia* of *Brugia malayi*. PLoS One 7:e51597. doi:10.1371/journal.pone.0051597. 23251587 PMC3518464

[98] Bauer E, Salem H, Marz M, Vogel H, Kaltenpoth M. 2014. Transcriptomic immune response of the cotton stainer *Dysdercus fasciatus* to experimental elimination of vitamin-supplementing intestinal symbionts. PLoS One 9:e114865. doi:10.1371/journal.pone.0114865. 25490201 PMC4260922

[99] Salem H, Bauer E, Strauss AS, Vogel H, Marz M, Kaltenpoth M. 2014. Vitamin supplementation by gut symbionts ensures metabolic homeostasis in an insect host. Proc R Soc B 281:20141838. doi:10.1098/rspb.2014.1838. PMC4213650 25339726

[100] Smith T, Driscoll T, Gillespie JJ, Raghavan R. 2015. A *Coxiella*-like endosymbiontis a potential vitamin source for the lone star tick. Genome Biol Evol 7:831–838. doi:10.1093/gbe/evv016. 25618142 PMC4994718

[101] Duron O, Morel O, Noël V, Buysse M, Binetruy F, Lancelot R, Loire E, Ménard C, Bouchez O, Vavre F, Vial L. 2018. Tick-bacterium mutualism depends on B vitamin synthesis pathways. Curr Biol 28:1896–1902. doi:10.1016/j.cub.2018.04.038. 29861133

[102] Smith E, Hoi JT, Eissenberg JC, Shoemaker JD, Neckameyer WS, Ilvarsonn AM, Harshman LG, Schlegel VL, Zempleni J. 2007. Feeding *Drosophila* a biotin-deficient diet for multiple generations increases stress resistance and lifespan and alters gene expression and histone biotinylation patterns. J Nutr 137:2006–2012. doi:10.1093/jn/137.9.2006. 17709434 PMC2196439

[103] Camporeale G, Giordano E, Rendina R, Zempleni J, Eissenberg JC. 2006. *Drosophila melanogaster* holocarboxylase synthetase is a chromosomal protein required for normal histone biotinylation, gene transcription patterns, lifespan, and heat tolerance. J Nutr 136:2735–2742. doi:10.1093/jn/136.11.2735. 17056793 PMC1626655

[104] Matthews MK, Wilcox H, Hughes R, Veloz M, Hammer A, Banks B, Walters A, Schneider KJ, Sexton CE, Chaston JM. 2020. Genetic influences of the microbiota on the lifespan of *Drosophila melanogaster*. Appl Environ Microbiol 86:e00305-20. doi:10.1128/AEM.00305-20. 32144104 PMC7205492

